# Phenotypic Plasticity Conferred by the Metastatic Microenvironment of the Brain Strengthens the Intracranial Tumorigenicity of Lung Tumor Cells

**DOI:** 10.3389/fonc.2021.637911

**Published:** 2021-05-13

**Authors:** Xu-Ge Wei, Ke-Wei Bi, Bo Li

**Affiliations:** Key Laboratory of Cell Biology, Department of Developmental Cell Biology, Ministry of Public Health and Key Laboratory of Medical Cell Biology, Ministry of Education, China Medical University, Shenyang, China

**Keywords:** HOXB9, phenotypic plasticity, brain metastatic microenvironment, lung cancer, cancer metastasis

## Abstract

Lung cancer is the leading cause of cancer-related deaths and is the primary source of brain metastases. Despite great advances in the study of the genetics and etiology of lung cancer in previous decades, the identification of the factors and mechanisms underlying the brain metastasis of lung tumors is still an open question. In this study, the results of bioinformatic conjoint analysis revealed that the metastatic microenvironment in the brain conferred lung tumor cell phenotypic plasticity, characterized by neural cell-like and embryonic–stem cell-like features. Meanwhile, the metabolic phenotype of the educated tumor cells underwent transition characterized by oxygen-related metabolism. The results of the experiments demonstrated that the downregulation of HOXB9 weakened the tumorigenicity of lung tumor cells. Bioinformatic prediction analysis also determined that many cell cycle-associated factors were potentially transcribed by HOXB9. Collectively, the results of this study suggested that under the influence of the metastatic environment of the brain, lung tumor cells seemed to acquire phenotypic plasticity characterized by neural cell-like features, and this transition may be associated with the aberrant upregulation of HOXB9.

## Introduction

Lung cancer is the primary cause of cancer-related deaths and is the main source of brain metastases (1–5). Brain metastasis severely impairs the survival and life quality of patients (6). Despite great progress in previous decades in the treatment of patients with lung cancer, the prognosis of patients with brain metastases to date remains poor. Thus, the exploration of the unknown genetic and molecular factors underlying the metastasis of tumor cells is urgently needed. The branched evolution of metastatic tumor cells influenced by the particular metastatic microenvironment and heterogeneity increases the difficulty of conducting such studies (7, 8). In addition to the divergence of gene expression signatures between brain metastases and primary tumors, this dissimilarity also presented in the comparison of brain metastases between regional lymph nodes and other extracranial metastases (7). The remarkable alterations may have a strong correlation with the unique intracranial tumor microenvironment (TME) since studies showed that brain metastases manifested with neural cell-like (NC-like) features (9, 10). The phenotypic transition has also been exhibited by different types of tumors (11). These findings suggest that the metastatic environment of the brain in particular favors the genetic alteration and expression profile modulation of tumor cells (12), which is characterized by the transition of the tumorous phenotype. Thus, a conjoint strategy of analysis and experiments may be needed to explore the common genes or mechanisms underlying the brain metastasis of different tumors. The aim of this study was to explore the common factors and mechanisms adopted by different subtypes of lung tumor to adapt to the unique microenvironment in the brain. Therefore, conjoint analysis using datasets of non-small cell lung cancer (NSCLC) and small cell lung cancer (SCLC) was performed.

After reaching the microvessels in the brain, tumor cells are typically intravascular for several days, during which the tumor cells modulate their cellular shapes to adapt to the narrowed lumen. The crosstalk between vessel endothelial cells (ECs) and metastatic tumor cells during the intravascular period and post-extravasation is still an unexplored field of research (13). ECs are essential components of trios, including the blood-brain-barrier (BBB), neurovascular unit, and brain metastatic niche. At the same time, ECs are the first type of cells that metastatic tumor cells encounter during extravasation and subsequential colonization in the brain. Consequently, it is reasonable to speculate that the communication between ECs and tumor cells plays a key role during the metastasis and colonization of tumor cells. As such, the alterations of junction-related genes in ECs impact the permeability of the BBB, which influence the metastasis of lung cancer and the activation of STAT3 in ECs, enabling tumor cell metastasis (14, 15). On the other hand, ECs serve as niche cells supporting the growth of tumor cells (16). However, the influence of ECs on the expression profile of tumor cells is still poorly characterized.

In this study, we adopted the strategy of conjoint analyses and experiments to explore the common genes and mechanisms underlying the brain metastasis of different subtypes of lung tumors. We analyzed the signature of the transcriptome of human SCLC tumor cell NCI-H209 that was co-cultured with human brain microvascular endothelial cells (HBMECs) and reanalyzed the transcriptome data from the public accessible gene expression omnibus (GEO) database. The results revealed that under the influence of the metastatic microenvironment of the brain, lung tumor cells underwent phenotypic transition characterized by NC-like and embryonic–stem cell-like (ESC-like) features. The *in vivo*/*vitro* experiments determined that HOXB9 participated in the brain metastasis of lung tumor cells.

## Materials and Methods

### Cell Culture

The HBMECs (Johns Hopkins University School of Medicine, Baltimore, MD, USA) were cultured in RPMI 1640 medium, which contained 10% fetal bovine serum (FBS; Thermo Fisher Scientific, MA, USA) and 10% Nu-serum (BD Biosciences, NJ, USA). The human SCLC tumor cell line NCI-H209 and human NSCLC cell line A549 were acquired from the American Type Culture Collection (Rockville, MD, USA). NCI-H209 cells were grown in RPMI 1640 medium, which contained 10% FBS. A549 cells were maintained in Dulbecco’s Modified Eagle’s medium supplemented with 10% FBS. All of the cells were incubated in 5% CO_2_ at 37°C.

### *In Vitro* Co-Culture of NCI-H209 Tumor Cells With HBMECs

5 × 10^5^ HBMECs were plated in the upper chamber of the Transwell insert with a 0.4-μm pore size (Corning Costar, Cambridge, MA, USA), then incubated in an incubator with a humidified atmosphere with 5% CO_2_ for 12 h. The same amounts of NCI-H209 cells that were seeded in the 6-well plate underwent incubation in the same conditions as that of the HBMECs. After incubation for 12 h, the Transwell insert containing the HBMECs was loaded in the 6-well plate with NCI-H209 followed by co-culture for 24 h, and the NCI-H209 cells were collected for the following experiments.

### Transcriptomic Sequencing of Co-Cultured NCI-H209

The co-cultured NCI-H209 cells were collected in Trizol (Takara Bio, Shiga, Japan), which followed the subsequential operation under the instruction of the technology company of Novegene.

### Raw RNA-Seq Data

The transcriptome data of NCI-H209 co-cultured with HBMEC was acquired *via* RNA-Seq analysis. The transcriptome data of brain metastatic PC9 and Lewis lung cancer (LLC) tumor cells were generated from the reanalysis of the raw sequencing data belonging to the subsets of the GSE83132 dataset in the GEO database.

### Transcriptome Data of Clinical Samples and the Corresponding Clinical Pathological Data

Transcriptome RNA-Seq data of lung adenocarcinoma (LUAD) (551 cases comprising 54 normal samples and 497 tumor samples) and the corresponding clinical data were downloaded from the third level of The Cancer Genome Atlas (TCGA) database (https://portal.gdc.cancer.gov/).

### Establishment of Differential Expression Genes (DEGs) in Three Datasets

Three sets of DEGs in the three datasets (NCI-H209, PC9, and LLC) were established by the comparison of the experimental groups and control groups, during which the parental tumor cell lines were designated as the control group (parental NCI-H209 tumor cells as the control group in the NCI-H209 dataset, parental PC9 tumor cells and parental LLC tumor cells as the control groups in the PC9 and LLC datasets, respectively) and the tumor cells that underwent experiments were regarded as the experimental group (NCI-H209 tumor cells co-cultured with HBMECs as the experimental group in the NCI-H209 dataset, brain metastatic PC9 and LLC tumor cells as the experimental groups in the PC9 and LLC datasets, respectively). The absolute value of the fold change >1 and the corresponding *P* values < 0.05 were regarded as the DEGs.

### GO and KEGG Signaling Pathways Enrichment Analysis

Three sets of DEGs deriving from the respective three datasets were used for the enrichment analysis. R language loaded with packages of clusterProfiler, enrichplot, and ggplot2 was used for enrichment analysis. The enriched terms with *p-* and *q-*values <0.05 were considered significant.

### Analysis of Semantic Similarity

The R package of GOSemSim was mainly used to perform the analysis of semantic similarity of the nine common genes and to calculate the similarity between terms of the Biological Process (BP) category co-enriched by co-cultured NCI-H209 and brain metastatic PC9.

### Correlation Analysis of Common Nine Genes Expression

Pearson coefficient was used to measures the correlation between genes and only *p*-values < 0.05 were considered significant.

### Analysis of Protein-Interaction Coupling Correlation

Based on the interaction of proteins recorded in the National Center for Biotechnology Information gene database, the hypergeometric test was used to calculate the *p*-value of the coupling correlation, which underwent transformation of false discovery rate.

### Gene Set Enrichment Analysis (GSEA)

The software gsea-3.0 was utilized to perform the GSEA, in which the gene set of HALLMARK in Broad Institute was used as the targeted gene set. All genes expressed were used for GSEA, and gene sets with NOM *p*- and *q*-values <0.05 were considered significantly enriched.

### Establishment of the Mouse Model of Brain Metastasis of Lung Cancer

Tumor cells (Lewis lung carcinoma cell line, LLC), with a density of 2 de^5^/100 µL and a volume of 100 µL, were injected into the internal carotid artery (ICA) of 6-week-old C57 mice. All of the study work and surgery protocols carried out on the mice were conducted in accordance with the guidelines of the institution where the study took place and approved by the Animal Care and Use Committee of China Medical University. The brain metastases were dissociated and harvested after the sacrifice of the mice, after which the metastatic cells underwent the minimum culture that was subsequently used for the next experiments.

### Transfection of Tumor Cells With Recombinant Lentivirus Particles Containing HOXB9 shRNA

The tumor cells were transfected with lentivirus particles containing HOXB9 shRNA (NM_008270, Target sequence TCAATCTGAGTGAGAGACA) following the instruction of the manufacturer (Shanghai Genechem Company, Shanghai, China), after which clones of the tumor cells positively transfected with the recombinant lentivirus were purified by puromycin.

### RNA Isolation and Real-Time Polymerase Chain Reaction (PCR)

The total RNA was isolated with Trizol (Takara Bio, Shiga, Japan) and reversed transcription was performed under the instruction of PrimeScript™ RT reagent Kit (Takara Bio, Shiga, Japan). The real-time PCR was operated by the ABI 7500 (Applied BioSystems, CA, USA) with the TB Green^@^ Premix DimerEraser™ (Takara Bio, Shiga, Japan) following the instructions of the manufacturer. The primers for HOXB9 and ACTB were as follows: HOXB9 Forward CGAGTACAGTTTGGAAACTTCG; Reverse CGCAAATTTTATTGTCCCCGTA; ACTB mouse (purchased from Sangon Biotech NO. B662302); and ACTB human (Sangon Biotech NO. B662102). The expression level was normalized to that of the ACTB serving as the internal control.

### Western Blot

The cells were lysed on ice and submitted to the western blot; the details are described in a previous study (17). The primary antibodies used in this study were listed as follows: HOXB9 (1:1000; Thermo Fisher Scientific, MA, US); β-tubulin (1:1000 dilution; Wanleibio Co., Ltd., Shenyang, China); HRP-conjugated antibodies (1:10,000; Santa Cruz Biotechnology, Inc., TX, USA).

### Immunofluorescence

The cells of LLC-brain metastatic tumor (BrM) were seeded on coverslips, followed by incubation for 12 h at 37°C. The cells were fixed with 4% paraformaldehyde and then treated with 0.2% Triton X-100 for permeabilization, after which 5% bovine serum albumin in phosphate-buffered saline (PBS) was used for blocking. The cells were stained with antibodies against Cytokeratin 19 (dilution of 1:100; Santa Cruz Biotechnology, Inc., TX, USA) and then incubated with a secondary antibody conjugated with Alexa-fluorescence 594 (1:200 Invitrogen, CA, USA). After staining with 4′,6-diamidino-2-phenylindole, confocal laser scanning microscopy (LSM 880; Zeiss Co., Ltd., Oberkochen, Germany) was used for observation and analysis. In the immunofluorescence of the brain slice (50 μm) the primary antibodies used were as follows: HOXB9 (1:100 in dilution); CD31 (1:100; BD Biosciences, NJ, USA); Ki67 (1:100; Cell Signaling Technology, Inc., MA, USA).

### Cell Viability Assay

Tumor cells (2´10^3^) were seeded in the 96-well culture plate under the designated experimental conditions. The viability of cells was determined at the indicated time by the microplate reader with the CellTiter 96 AQueous One Solution Cell Proliferation Assay (MST assay; Promega, Madison, WI, USA).

### Flow Cytometry

The cells were plated in the 6-well plate and incubated for 24 h, after which cells were suspended in PBS and fixed in 70% ethanol at 4°C. The cells were then incubated in the solution of propidium and RNase for 30 min. The analysis of the cell cycle was performed using a flowcytometer (BD Accuri**^®^** C6; BD Biosciences, NJ, USA).

### Prediction Analysis of Cell-Cycle-Associated Genes Potentially Transcribed by HOXB9

The R packages TFBSTools and JASPAR2020 were used to perform the prediction analysis.

### Statistics

All quantitative variables were shown as mean ± standard deviation (SD), and the difference between both groups was determined by the Student’s t-test, in which *p*-values <0.05 were considered significant. GraphPad Prism 8.0 software was used for the statistical analyses.

## Results

### Strategy of Conjoint Analysis to Reveal the Common Genes Associated With the Brain Metastasis of Lung Tumor Cells

**Figure 1:**
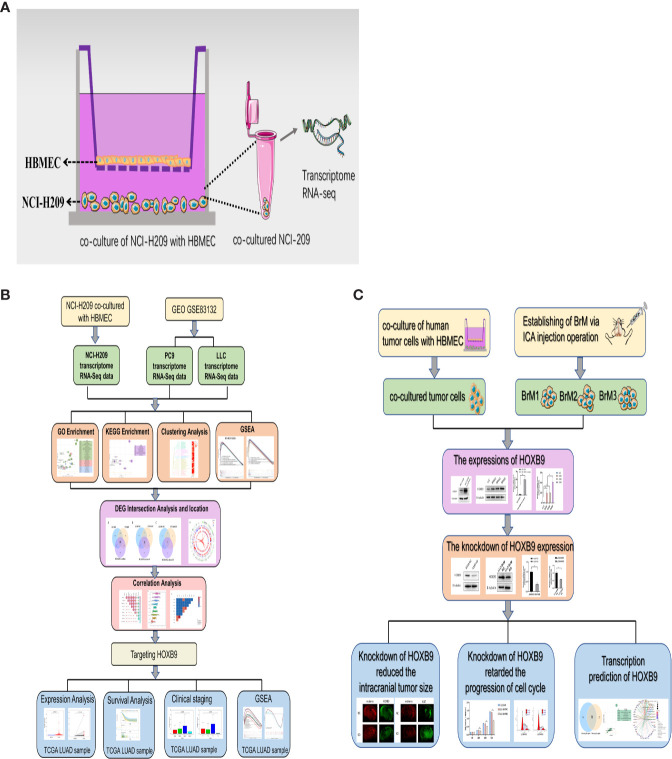
Workflow of this study. **(A)** Schematic diagram of the *in vitro* co-culture system. **(B)** Workflow process of the bioinformatic analysis. **(C)** Workflow of the experiments.

To explore the common factors and mechanisms underlying the brain metastasis of lung tumor cells, a strategy of conjoint analysis was developed, which included the dataset of co-cultured NCI-H209 tumor cells and datasets of the brain metastatic PC9 and LLC tumor cells. First, as shown in **Figure 1A**, to recapitulate the influence of the metastatic vascular niche on the tumor cells, NCI-H209 cells, the cell line of SCLC, were co-cultured with HBMECs for 24 h. The gene expression profile of the educated NCI-H209 cells was then analyzed (GSE157219, https://www.ncbi.nlm.nih.gov/geo/query/acc.cgi?acc=GSE157219). In addition, two sets of RNA-Seq original raw data derived from subsets of the GSE83132 dataset (https://www.ncbi.nlm.nih.gov/geo/query/acc.cgi?acc=GSE83132) in the GEO database were downloaded in order to profile the signature of lung tumor cells that metastasized to the brain. Here, the lung tumor cells derived from humans and mice were injected into the ventricles of the mouse followed by the gene expression analysis of the brain metastases. The comparative analysis based on the comparison of the experimental tumors between the control tumors revealed the common genes or mechanisms underlying the metastasis of lung tumor cells. The workflow of this study is shown in **Figures 1B, C**.

### Lung Tumor Cells Acquired Phenotypic Plasticity Characterized by NC-Like Features Under the Influence of Brain Metastatic Microenvironment

**Figure 2:**
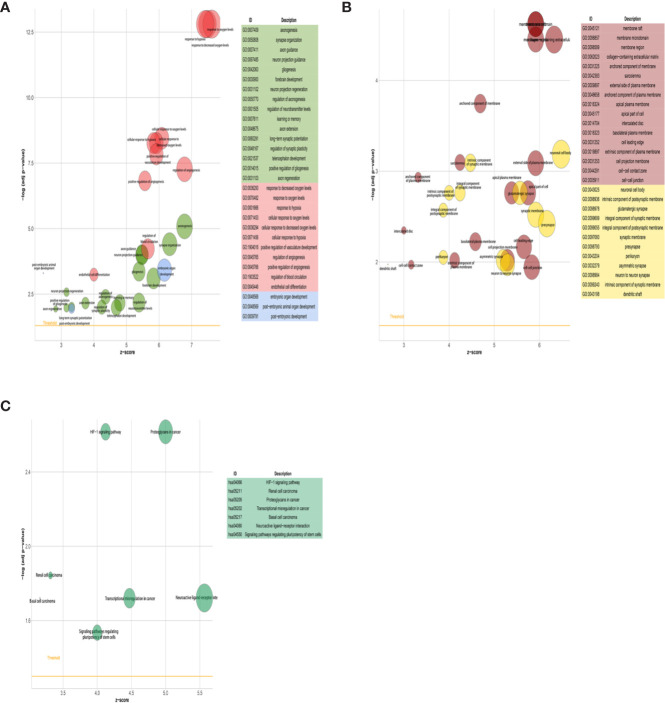
GO and KEGG signaling pathways enrichment analysis of NCI-H209 co-cultured with HBMECs. **(A, B)** Enrichment analysis of BP and CC categories in GO database. **(C)** Signaling pathways in KEGG database enrichment analysis. The *p*- and *q*-values of the enriched terms as shown in the plot were both <0.05.

**Figure 3:**
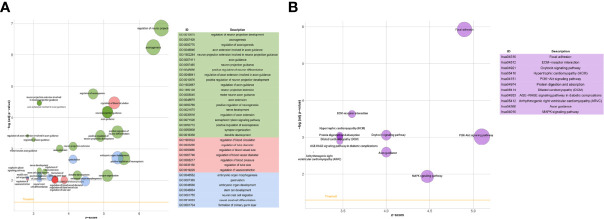
GO and KEGG signaling pathways enrichment analysis of brain metastatic PC9 tumor cells. **(A)** BP category in GO database enrichment analysis. **(B)** Signaling pathways in KEGG database enrichment analysis.

**Figure 4:**
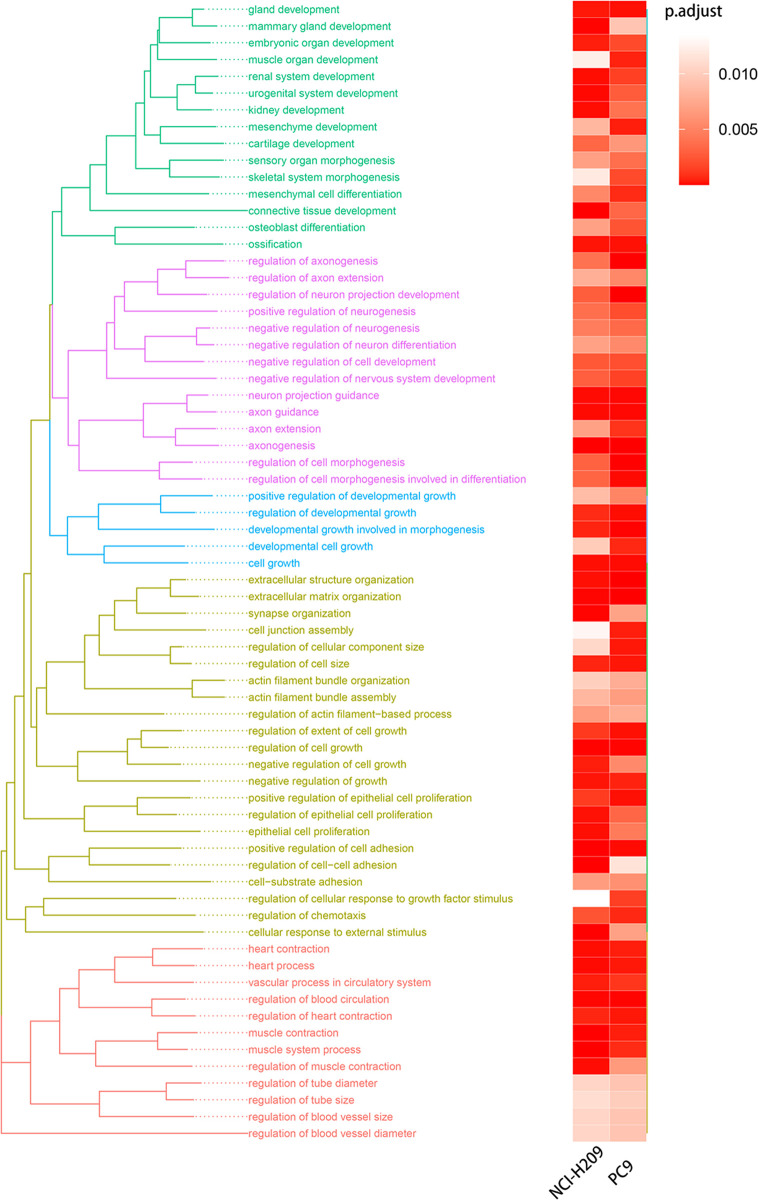
Semantic similarity clustering analysis. The terms of BP co-enriched significantly by the co-cultured NCI-H209 cells and brain metastatic PC9 were used for clustering analysis.

We first compared the gene expression profile between the experimental and control groups. The DEGs were acquired from three sets of RNA-Seq data, termed NCI-H209 DEG set, PC9 DEG set, and LLC DEG set, respectively. To determine the positively vibrant biological activities, the upregulated DEGs from the three aforementioned datasets were used to perform enrichment analyses, including BP and cellular component categories in the gene ontology (GO) database and signaling pathways in the Kyoto Encyclopedia of Genes and Genomes (KEGG) database, respectively. Positively enriched GO terms and KEGG pathways by upregulated DEGs in the NCI-H209 DEG set are shown in **Figure 2**. The positively enriched terms represented the features of neural cells, such as axonogenesis, neuron projection guidance (**Figure 2A**), neural cell body (**Figure 2B**), and neuroactive ligand-receptor interaction (**Figure 2C**). In addition to the terms unique to neural cells, terms associated with the embryo development were positively active, such as embryonic organ development, post-embryonic development, and signaling pathways regulating pluripotency of stem cells (**Figures 2A, C**). The hypoxia-inducible factor 1 signaling pathway was also activated in NCI-H209 cells co-cultured with brain microvascular ECs (**Figure 2C**), which suggested that there was a modulation of oxygen homeostasis. Furthermore, enrichment analysis of the upregulated DEGs in the PC9 DEG set utilizing the BP category and signaling pathways in GO and KEGG database showed that these positively enriched terms also represented the features of neural cells, such as regulation of neuron projection development, axonogenesis (**Figure 3A**), and axon guidance (**Figure 3B**). These results suggested that lung cancer cells seemed to acquire NC-like phenotypic characteristics coupled with the regulation of response to oxygen during brain metastasis. Upregulated DEGs in the LLC DEG set were mainly involved in cellular energy metabolism and DNA replication and transcription, which indicated that beyond the NC-like phenotypic transition, the proliferation capability was also positively enhanced (**Supplementary Figure 1**). Semantic similarity clustering analysis was performed toward the co-enriched terms by NCI-H209 and PC9 DEG sets annotated by the BP category in the GO database. As shown in **Figure 4**, the positively enriched terms clustered toward characterizing NC-like cells according to their biological function, including regulation of nervous system development, axonogenesis, and neuron differentiation. These results suggested that lung cancer cells acquired phenotypic plasticity characterized by NC-like features under the influence of the microenvironment of brain metastasis. Meanwhile, the terms referring to cell metabolism and growth were also obviously enriched, which suggested that metastatic lung tumor cells acquired more survivability.

### Identification of the Genes Associated With the Phenotypic Transition of Lung Tumor Cells

**Figure 5:**
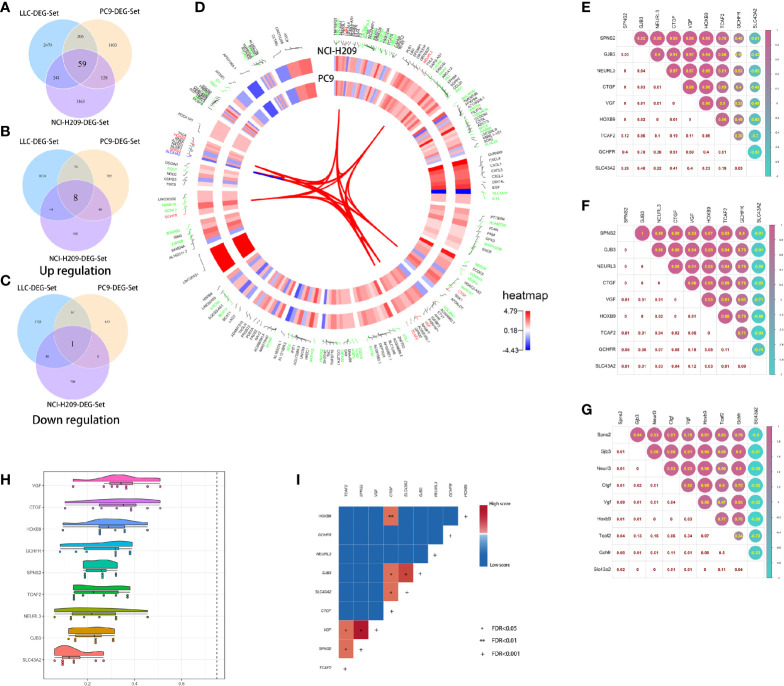
Correlation analysis of the common DEGs. **(A)** The intersection of three DEG sets. **(B)** The eight common upregulated DEGs shared by three DEG sets. **(C)** The common downregulated DEG shared by three DEG sets. **(D)** The chromosome location of the common DEGs shared by both DEG sets of co-cultured NCI-H209 and brain metastatic PC9. **(E)** The expression correlation analysis of the nine common genes in the co-cultured NCI-H209 cells. **(F)** The expression correlation analysis of the nine common genes in the brain metastatic PC9 cells. **(G)** The expression correlation analysis of the nine common genes in the brain metastatic LLC cells. **(H)** Semantic similarity analysis of the nine common genes. **(I)** The protein-interaction coupling correlation analysis of the nine common genes, *FDR < 0.05, **FDR < 0.01, +FDR < 0.001.

The above results suggested that the brain metastatic lung tumor cells exhibited some NC-like features and that they were reprogrammed to acquire an NC-like phenotype. This phenotypic plasticity might be a prerequisite for successful colonization of the brain metastatic lung tumor cells. To identify the driving genes during this process, the intersection analysis toward three DEG sets was performed and 59 DEGs were identified (**Figure 5A**). Nine out of the 59 DEGs had consistent expression tropism across three DEG sets. Eight out of the nine (HOXB9, GCHFR, NEURL3, GJB3, CTGF, VGF, SPNS2, and TCAF2) were upregulated in three DEG sets (**Figure 5B**) and only one was downregulated (**Figure 5C**). Interestingly, some of the nine genes are abundant in the central nervous system (CNS). For example, CTGF, known as the sub-plate neurons marker, plays an important role during brain development and cortical maturation; GCHFR may play a role in the production of biogenic amine neurotransmitters in brain 5-hydroxytryptamine neurons. VGF was originally identified in the neurons and neuroendocrine cells and is responsible for neurogenesis and neuroplasticity associated with learning, memory, depression, and chronic pain; HOXB9, as a member of the homeobox family, plays a fundamental role in determining positional identity along the anterior-posterior body axis during embryogenesis. Collectively, lung tumor cells educated by the brain microenvironment seem to acquire phenotypic plasticity by the upregulated expression of CNS-relevant genes which strengthened the tumorigenicity of the tumor cells. The underlying correlation between these genes was determined by several analyses, including chromosome localization, expression correlation, semantic similarity, and interaction coupling. As shown in **Figure 5D**, among the nine common genes shared by three DEG sets, HOXB9, SPNS2, and SLC43A2, and VGF and TCAF2 are co-located in the same chromosomes, respectively, which may underlie the existence of co-expression of genes. Furthermore, there were expression correlations between the nine genes. Eight out of nine had reciprocal positive expression correlation and one gene, SLC43A2, held negative correlations with the others (**Figures 5E–G**). The results of semantic similarity analysis suggested that the nine genes had relatively lower similarity, which implied the roles they play in multiple biological processes (**Figure 5H**). Despite the relative lower similarity in semantics, the nine genes showed obvious pertinence in molecular function. Therefore, they may have some potential direct or indirect interaction. In order to further explore the underlying correlation between the nine genes, the analysis of protein interaction coupling correlation was performed. The results showed significant associations between pairs of candidate genes (**Figure 5I**). Taken together, these results suggested the existence of unique driver genes associated with the phenotypic transition of brain metastatic lung tumor cells.

### HOXB9 and CTGF Served as Biomarkers for Brain Metastatic Lung Tumor Cells

**Figure 6:**
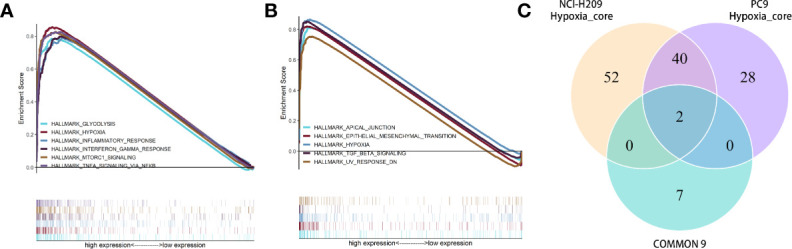
GSEA toward the whole expressed genes in the cells of co-cultured NCI-H209 and brain metastatic PC9. **(A)** GSEA for the NCI-H209 cells co-cultured with HBMECs. **(B)** GSEA for the whole expressed genes in the brain metastatic PC9 cells. The gene sets with *p*- and *q*-values <0.05 were considered significantly enriched. **(C)** Intersection analysis towards three gene sets, including two hypoxia gene sets (respectively enriched in co-cultured NCI-H209 and brain metastatic PC9) and the nine common genes.

In order to exclude the subjective factors introduced by setting an artificial threshold to define DEGs, GSEA was performed. As shown by the results, gene sets associated with oxygen-related metabolism, such as “hypoxia”, “glycolysis”, “oxidative phosphorylation”, and “reactive oxygen species pathway”, were significantly enriched by brain metastatic lung tumor cells (**Figures 6A, B**, **Supplementary Figure 1E**, and **Supplementary Tables 1–3**). The transcriptomic features manifested by the upregulated DEGs drawn respectively from three datasets seemed to be similar, which suggested that the upregulated DEG subsets were highly correlated with the phenotypes of lung tumor cells under the influence of the microenvironment of brain metastasis. Intriguingly, gene set “hypoxia” was simultaneously enriched in NCI-H209 cells co-cultured with HBMECs and brain metastatic PC9 cells. These findings provided a clue that the genes involved in the hypoxia response pathway might confer the phenotypic plasticity of brain metastatic lung cancer cells. Therefore, we performed the intersection analysis with nine genes and two sets of core genes extracted from hypoxia gene subsets that were co-enriched by co-cultured NCI-H209 cells and brain metastatic PC9 cells, respectively. Finally, only two genes, HOXB9 and CTGF, were shared by three sets of genes (**Figure 6C**), which suggested that they may be biomarkers for brain metastatic lung tumor cells.

### Downregulation of HOXB9 Weakened the Intracranial Tumorigenicity of Lung Tumor Cells

**Figure 7:**
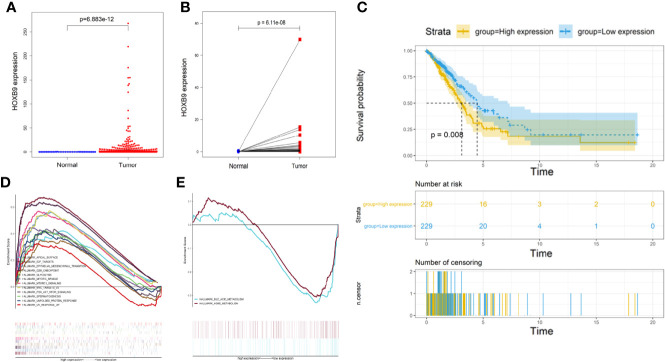
Analysis of differential expression, survival, and GSEA of HOXB9. **(A)** The differential expression of HOXB9 between the overall normal and tumor tissues in the LUAD patients in TCGA database, *p* < 0.05. **(B)** The differential expression of HOXB9 between the normal and tumor tissues deriving from the same patient, *p* < 0.05. **(C)** Survival analysis of HOXB9 for the LUAD patients from TCGA database, *p* < 0.05. **(D)** The significantly enriched gene sets by the samples of HOXB9 upregulation in the GSEA. **(E)** Enriched gene sets by the samples with HOXB9 downregulation in the GSEA. Terms of the gene sets enriched with *p*-and *q*-values < 0.05 were considered significantly enriched.

**Figure 8:**
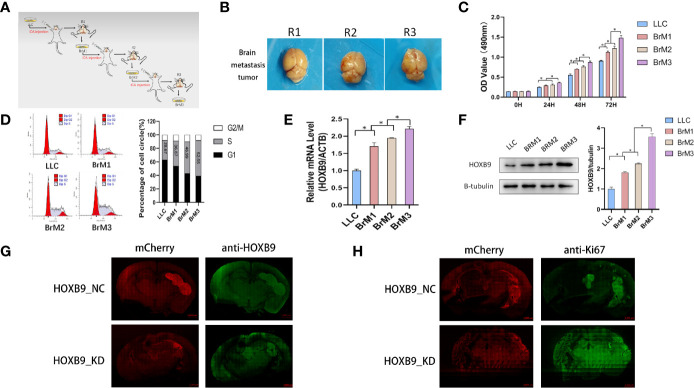
Downregulation of HOXB9 decreased the intracranial tumorigenicity of the brain metastatic lung tumors. **(A)** The schematic diagram of establishing the mouse model of brain metastasis of lung tumor. **(B)** The brain metastatic tumors in the three rounds of modeling *via* ICA injection. R1: the first round of modeling *via* ICA injection; R2: the second round of modeling; R3: the third round of modeling. **(C)** Cell viability analysis of the brain metastatic tumor cells, derived from three rounds of mouse models and the parental LLC cells, BrM1: brain metastatic tumor 1, deriving from the mouse model in first round of modeling, R1; BrM2: brain metastatic tumor 2, deriving from R2; BrM3: brain metastatic tumor 3, from R3. *p < 0.05. **(D)** Flow cytometry analysis for the cell cycle progression of BrMs and parental LLC cells. **(E)** Determination of HOXB9 expression in the level of mRNA *via* real-time PCR, *p < 0.05. **(F)** Western blot analysis for the expression of HOXB9, *p < 0.05. **(G)** The downregulation of HOXB9 in LLC-BrM (also indicated as BrM3) decreased the size of intracranial colonization, the red fluorescence mCherry indicated the location of intracranial metastases, and the green fluorescent anti-HOXB9 denoted the expression of HOXB9, scale bar 1000 μm. **(H)** Downregulation of HOXB9 decreased the expression of Ki67 in LLC-BrM, the green fluorescence indicated the expression of Ki67. Scale bar indicating 1000 μm.

Studies showed some embryonic development-related genes that were conserved in the evolution were involved in the progression of tumors, such as SOX9, GATA-4/6, Snail/Slug, and Six1. Thus, we decided to focus on HOXB9. The transcriptomic RNA-Seq data of LUAD samples along with the corresponding clinicopathological data were downloaded from TCGA database (**Supplementary Table 4**). First, the expression levels of HOXB9 in total LUAD tumor samples were significantly elevated compared with the control groups (**Figure 7A**). In addition, the pairing analysis in which the normal and tumor samples were derived from the same patient further confirmed the differential expression of HOXB9 in the normal and tumor tissue (**Figure 7B**). Although the expression levels of HOXB9 in LUAD patients were not significantly correlated with the pathological staging (**Supplementary Figures 2A–D**), the survival analysis indicated that the upregulated expression of HOXB9 was significantly associated with the poor rate of survival of the patients (**Figure 7C**, *p* < 0.01). The results suggested that HOXB9 seemed to be a malignant marker for LUAD patients. In the following analysis, GSEA was performed to explore the gene expression signature of patients of HOXB9 upregulation and downregulation. The results suggested that tumors of higher expression of HOXB9 manifested with vibrant cell cycle activity as gene sets regarding cell cycle were enriched significantly (**Figure 7D**). By contrast, only two gene sets were enriched by the tumors of lower HOXB9 expression (**Figure 7E**). That may suggest that HOXB9 seems to be a marker indicating the acquirement of malignant phenotypes for lung tumor cells. Given that the brain microvascular ECs were the first kind of cells that interacted with the metastatic tumor cells extravasating from the vessels, we performed a co-culture experiment to determine the expression modulation of HOXB9 in lung tumor cells educated by HBMECs. As shown in **Supplementary Figure 3**, the levels of HOXB9 in NCI-H209 cells and A549 cells were significantly increased after being co-cultured, which suggested that the initial signal to trigger HOXB9 expression may be derived from the brain microvascular niche. To further determine whether HOXB9 was required by lung tumor cells during brain metastasis, we established the mouse model of extracranial tumor metastasis. First, the parental LLC cells, a LUAD cell line of the mouse, were transported into the brain of a C57 mouse *via* the operation of ICA injection to produce brain metastases. After the mouse bearing brain metastases were euthanized, the brain metastases were dissociated and underwent minimum culture to produce the brain metastatic populations (brain metastatic tumor 1, BrM1). Then, the cells of BrM1 were re-injected into the brains of mice in the second round of ICA injection, which yielded BrM2 cell populations (brain metastatic tumor 2, BrM2), and the BrM3 cell population (brain metastatic tumor 3, BrM3; also referred to as LLC-BrM) was produced in the third round of ICA injection using the same method on the basis of BrM2 (**Figure 8A**). The purity of BrMs was verified by the detection of cytokeratin 19, a tumor marker for lung cancer (**Supplementary Figure 4A**). As colonization in the brain was repeated, under the iterative education of the intracranial microenvironment, the intracranial tumorigenicity of BrM cells was dramatically enhanced (**Figures 8B, C**). This increasing proliferative capability of BrM cells to some extent was attributed to the rising proportion of tumor cells in the S phase (**Figure 8D**). In addition, the survival status statistics of the post-operative mice seemed to provide a clue to some extent that the iterative influence of the intracranial microenvironment the brain metastatic tumor cells acquired strengthened the capability of extravasation and proliferation because of the observation of the rising rate of weight loss and the decreasing percentage of hemiplegia of the mice injected with different BrMs (**Supplementary Figures 4B, C**). However, this correlation between the physiological status alteration of operative mice and the extravasation capability of the metastatic tumor cells still needed more experiments for validation. The expression levels of HOXB9 in cells of BrMs were then determined by experiments of qRT-PCR and western blot. As shown in **Figures 8E, F**, there was an increasing trend for the expression level of HOXB9 in different cells of brain metastatic BrMs. These results suggested that the upregulation of HOXB9 in brain metastatic tumors may be an adaptive response to the brain microenvironment. To further determine the roles of HOXB9, we transfected LLC-BrM cells with the mcherry-tagged lentiviral particles containing HOXB9 shRNA. The cells that transfected with non-silence shRNA were used as the controls. The results of qRT-PCR and Western blot indicated that the expression of HOXB9 was knocked down in cells of LLC-BrM (**Supplementary Figure 5A**). Next, the red fluorescence-labeled LLC-BrM cells were transferred into mice *via* ICA injection to establish the models of brain metastases (**Supplementary Figures 5B, C**). The results showed that the expression of HOXB9 was positively observed in the bulk of metastatic loci and the depletion of HOXB9 in cells of LLC-BrM could attenuate the colonization and survivability of tumor cells in the brain (**Figure 8G**). Simultaneously, all the brain metastases were almost significantly stained with Ki67, an intrinsic marker of cell proliferation, and the knockdown of HOXB9 led to the reduction of Ki67 in these loci (**Figure 8H**). Collectively, these findings suggested that the brain metastatic tumor cells may have upregulated the expression level of HOXB9, which strengthened the intracranial tumorigenicity.

### Upregulation of HOXB9 Promoted the G1/S Cell Cycle Transition in Brain Metastatic Lung Tumor Cells

**Figure 9:**
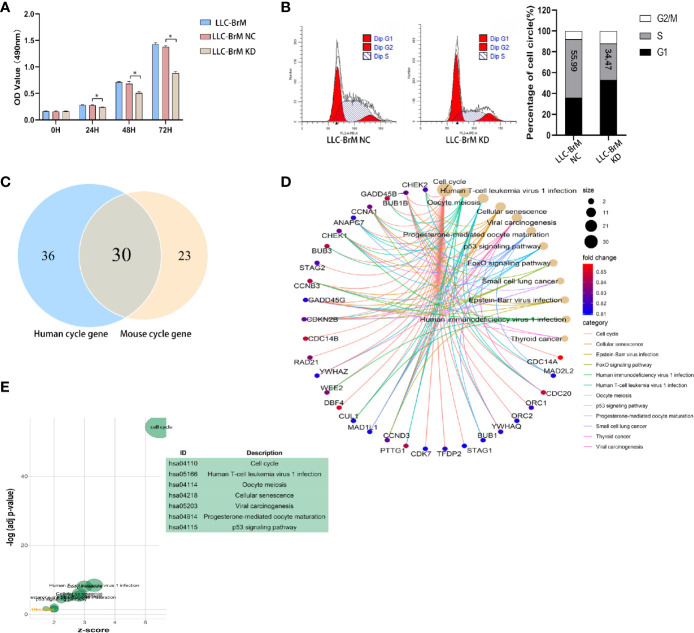
HOXB9 promoted the proliferation of brain metastatic tumor cells through promoting the G1/S transition of the cell cycle. **(A)** Downregulation of HOXB9 in LLC-BrM decreased the proliferation, *p < 0.05. **(B)** Flow cytometry analysis determined the decreasing proportion of cells in the S phase of the cell cycle as the downregulation of HOXB9 in LLC-BrM. **(C)** Intersection analysis established the common cell-cycle-associated genes potentially transcribed by HOXB9 (Hoxb9) shared by both human and mouse. **(D)** Enrichment analysis of BP category in GO database for the 30 common genes potentially transcribed by HOXB9, terms enriched with *p*- and *q*-values were <0.05. **(E)** Enrichment analysis with signaling pathways in KEGG database for the 30 common cell cycle-associated genes. The *p*- and *q*-values of these enriched terms were both <0.05.

The results of GSEA and enrichment analysis toward the LLC DEG set revealed that pathways and activities involved in the cell cycle, particularly the DNA replication, were vibrantly active (**Supplementary Figure 1**), which partly explains the *in vivo* experimental results described above. As shown in **Figure 9A**, knockdown of HOXB9 significantly inhibited the proliferation of LLC-BrM cells. To determine whether the inhibition of cell proliferation was due to the altered process of the cell cycle, we carried out flow cytometry using PI staining. The results suggested that the knockdown of HOXB9 in LLC-BrM cells led to an obvious increase in the G1 phase and a corresponding decrease in the S phase. No significant change was observed at the G2/M phase (**Figure 9B**). Moreover, similar results were also observed in A549 cells after transfection with HOXB9 shRNA (**Supplementary Figure 6**). Considering the fact that HOXB9 was a highly conserved transcriptional factor and may be associated with the transition of the G1/S phase in the cell cycle, the predictive analysis was performed to determine the factors involved in the cell cycle progression that were potentially transcribed by HOXB9. The cell-cycle-associated factors potentially transcribed by HOXB9 in humans and mice are listed in **Supplementary Tables 5**, **6**. In total, 66 and 53 cell cycle-related genes were potentially transcribed by HOXB9 (Hoxb9) in humans and mice, respectively, among which 30 genes were shared by both (**Figure 9C**). The results of enrichment analysis with category of BP in the GO database and signaling pathways in the KEGG database suggested that these genes potentially transcribed by HOXB9 (Hoxb9) are mainly involved in the growth and division of cells. Terms such as cell cycle, oocyte meiosis, and progesterone-mediated oocyte maturation were significantly enriched (**Figures 9D, E**). To our knowledge, proteins encoded by the genes of GADD45B, GADD45G, and CDKN2B served as the cell growth regulators that controlled G1/S transition of mitotic cell cycle. Terms of multiple tumor-associated pathways were also significantly enriched, particularly the term of SCLC (**Figure 9E**). The results suggested that these genes potentially transcribed by HOXB9 (Hoxb9) might be the downstream effectors in the HOXB9-mediated G1/S transition of the cell cycle.

## Discussion

Beginning from the original “seed-soil” theory to the current theory of TME, the significance of TME has been broadly recognized. The influence of TME on tumor cells is embodied in multiple aspects, such as the inherited feature of genes, branched evolutions of cells, genetic expression signature, representation of cellular stemness, and the plasticity of phenotypes (7, 18). The HBMEC not only serves as one of the primary components for neurovascular units in the brain, but also constitutes brain metastatic TME. In addition, the HBMEC is still one of the cell types that metastatic tumor cells would encounter and interact with during the cascade of intravasation and extravasation when they manage to metastasize to the brain. Accordingly, the roles of HBMEC during brain metastasis could not go unnoticed.

Recent studies found that the proliferation and metastasis of tumor cells were highly correlated with anaerobic respiration, which potentially made glycolysis the main research field for cancer therapy (19–22). The results of this study were supplementary to the recent studies to some extent, since the co-cultured tumor cells seemed to undergo the transition of overall metabolic status characterized by the potentiated activities and pathways responding to hypoxia, blood supply, and angiogenesis. This was also the marked features of tumor metabolism. In addition, under the influence of HBMECs, the tumor cells seemed to undergo phenotypic transition characteristic of the positive enrichment of NC-related and ESC-related gene sets, which suggested that tumor cells acquired NC-like and ESC-like phenotypes. These results were also consistent with the results of recent studies that, in a particular environment, tumor cells would present distinct gene expression profiles similar to the original resident cells (9–12, 23). Moreover, results in this study also seemed to be in agreement with the findings that there was a certain common mechanism adopted by different brain metastases (11).

The results of this study seem to suggest that it was the acquired phenotype endowing tumor cells with the adaptability to the new microenvironment consisting of various intracranial cellular types, particularly the neuron and corresponding assistant cells, and this transition may be termed “neural-stem-cell-mimesis”, a process of simulation mirroring the phenotype of neurons and stem cells, similar to how wild animals have adopted the strategy of changing colors or postures to merge with the ambient environment in order to escape their predators. For tumor cells facing a new extracellular environment, the imperative thing they may need to do is to conceal themselves, since acting like the original resident cells may be a good strategy to escape local immune surveillance. As the iterative acting of TME, the extravasation capability of tumor cells was enhanced to a certain degree that may be demonstrated by the declining of the hemiplegia proportion of the mouse undergoing ICA injection. In the first round of ICA injection, the percentage of hemiplegia was as high as 40%, which to some extent could be attributed to the block of intracranial arteries caused by the injected tumor cells. In the second and third rounds, particularly in the third, the percentage of hemiplegia dropped to 10%, which means that after being injected into the intracranial arteries, the tumor cells extravasated in a short time, which may be one of the reasons leading to the decrease of hemiplegia for the operated mouse.

As one of the highly conserved transcription factors in the evolution of the organism, HOXB9 has crucial effects during the embryonic development, which is why its expression level was strictly regulated in adult organisms (24). Meanwhile, HOXB9 is a member of the transcription family possessing homeobox, which plays a significant role in embryonic development and tumorigenesis (24). The gene family of HOX is highly conserved in the vertebrate and plays important roles in many aspects of development (25). In breast tumors, the expression of HOXB9 was upregulated, which induced the factors associated with the fate of tumor cells promoting the progression and metastasis of the disease (26, 27). It was reported that the expression level of HOXB9 was a potential prognostic factor for LUAD since the upregulated HOXB9 was correlated with the advanced clinicopathological staging (28). Recent studies showed that HOXB9 was a critical transcription factor for the drug resistance of tumors, the expression silence of which would be very promising for ameliorating the resistance of tumor cells to the agent (29). However, some studies proposed a thoroughly opposite view, concluding that the downregulation of HOXB9 was correlated with the poor survival prognosis of patients bearing adenocarcinoma of the colon, ductal adenocarcinoma of the pancreas, and gastric carcinoma (30–32). Consequently, the roles of HOXB9 in tumorigenesis and disease progression seem to vary between different tumor types, and this indicated the complex roles and mechanisms underlying tumorigenesis and disease progression. In addition, the mechanism regarding regulation of HOXB9 in tumors is still an open question. In this study, we found that the expression of HOXB9 in tumor cells was upregulated under the education of the metastatic environment of the brain, which strengthened the intracranial tumorigenicity of the tumors. While the expression of HOXB9 in primary tumors was shown to be upregulated, it may be conjectured that during the tumorigenesis and progression of the disease, HOXB9 played a pro-tumor role and that this tumor-favored effect was further potentiated by the brain metastatic microenvironment.

Collectively, in this study, we found that under the influence of the metastatic microenvironment of the brain, the lung tumor cells underwent phenotypic transition characterized by NC-like and ESC-like features; thus, we may further term the adaptive transition “neural-stem cell-mimesis”. In addition, the tumor cells presented the status of glycolysis, and *via* this phenotypic transition, the tumor cells gained potential competence of escaping local immune surveillance and advantages of growth. Further analysis and experiments revealed that in brain metastatic lung tumor cells, HOXB9 played significant roles in the proliferation and cell cycle progression. These results can provide an additional perspective for the treatment of brain metastasis of lung cancer.

## Data Availability Statement

The raw data and experimental detail could be accessed in GSE157219 (https://www.ncbi.nlm.nih.gov/geo/query/acc.cgi?acc=GSE157219). The datasets of brain metastatic cells of PC9 and LLC could be accessed in GSE83132 (https://www.ncbi.nlm.nih.gov/geo/query/acc.cgi?acc=GSE83132). The RNA-Seq data and corresponding clinicopathological data of LUAD patients in TCGA could be accessed in the TCGA database (https://portal.gdc.cancer.gov).

## Ethics Statement

The animal study was reviewed and approved by Animal Care and Use Committee of China Medical University.

## Author Contributions

The design and conceptualization were performed by X-GW, K-WB, and BL. Data analysis and visualization were performed by X-GW and K-WB. Establishment of animal models and experiments were accomplished by X-GW and K-WB. The original writing and editing of this manuscript were accomplished by X-GW, K-WB, and BL. All authors contributed to the article and approved the submitted version.

## Funding

This study was supported by the National Natural Science Foundation of China (Grant Nos. 81672881 and 82073242).

## Conflict of Interest

The authors declare that the research was conducted in the absence of any commercial or financial relationships that could be construed as a potential conflict of interest.

## Abbreviations

HBMEC, human brain microvascular endothelial cell; EC, endothelial cell; NSCLC, non-small cell lung cancer; SCLC, small cell lung cancer; GEO, gene expression omnibus; ICA, internal carotid artery; BrM, brain metastatic tumor; LLC, Lewis lung cancer; LUAD, lung adenocarcinoma; DEG, differential expressed gene; GSEA, gene set enrichment analysis; TME, tumor microenvironment; BP, biological process; MF, molecular function; GO, gene ontology; KEGG, Kyoto Encyclopedia of Genes and Genomes; TCGA, The Cancer Genome Atlas Program; NC-like, Neural-cell-like; ESC-like, Embryonic-stem-cell-like; PBS, phosphate-buffered saline.
