# Supplementary material for: Phenotypic Plasticity Conferred by the Metastatic Microenvironment of the Brain Strengthens the Intracranial Tumorigenicity of Lung Tumor Cells
Source: Front Oncol. 2021 May 13;11:637911. doi: 10.3389/fonc.2021.637911 (PMC8155524; doi:10.3389/fonc.2021.637911)
Supplement: Supplementary file 1 [file DataSheet_1.pdf]

Supplementary Material

1 Supplementary Figures and Tables

1.1 Supplementary Figures

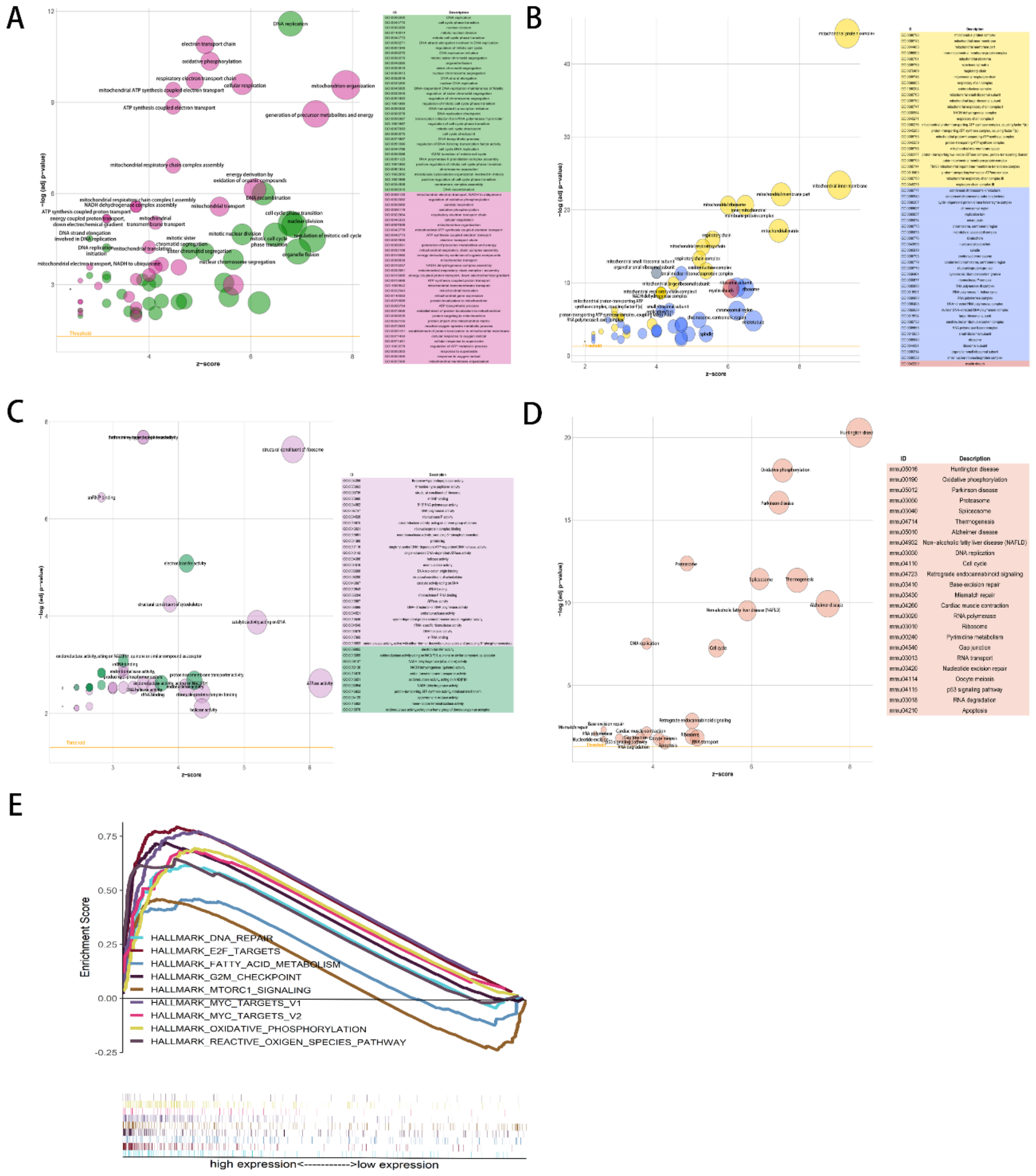

Supplementary Figure 1. Enrichment analysis of LLC dataset. GO and KEGG signaling pathways enrichment analysis utilized the upregulated DEGs in LLC DEG set, and GSEA used the whole expressed genes in brain metastatic LLC tumor cells. (A) Enrichment analysis of BP category in GO database with the upregulated DEGs in LLC DEG dataset. (B) Enrichment analysis of CC category. (C) Enrichment analysis of MF category in GO database. (D) Enrichment analysis of signaling pathways in KEGG database. Both *p*- and *q*-values of the factors shown in the plot were less than 0.05. (E) GSEA utilized the HALLMARK gene sets in the MSigDB with the whole expressed genes

in the brain metastatic LLC tumor cells. Only gene sets with *p*- and *q*-values both less than 0.05 were considered significantly enriched.

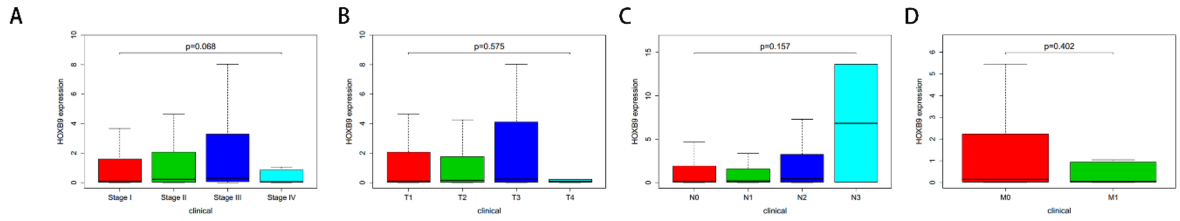

Supplementary Figure 2. Correlation analysis of HOXB9 expression with the clinicopathological features of LUAD patients in TCGA database. (A-D) Correlation analysis of HOXB9 expression with the stage and TNM of LUAD patients in TCGA database, $p = 0.068$, 0.575, 0.157, and 0.402, respectively.

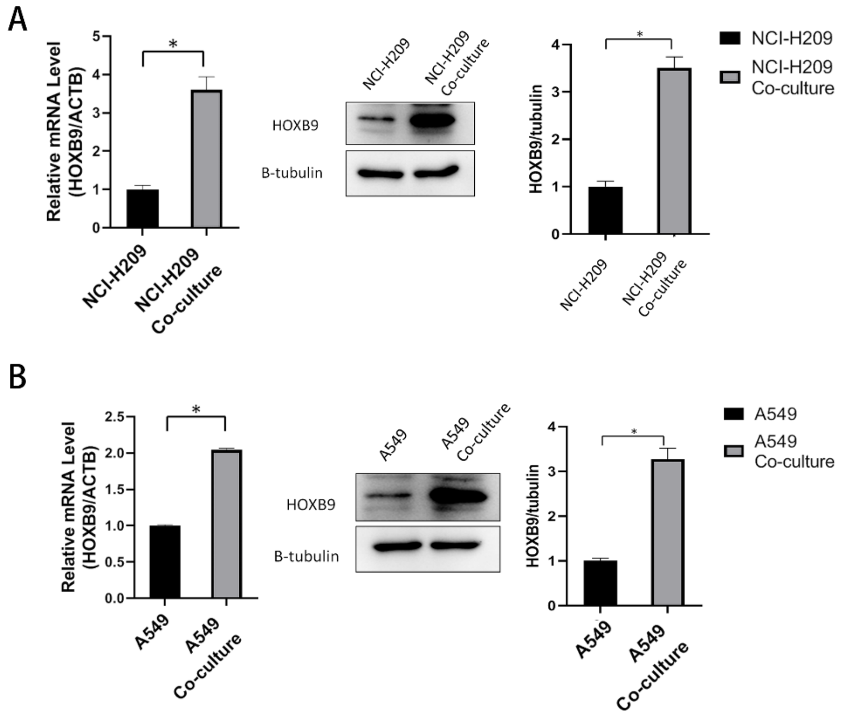

Supplementary Figure 3. Upregulation of HOXB9 in NCI-H209 and A549 co-cultured with HBMECs. (A) Upregulation of HOXB9 in the co-cultured NCI-H209 cells determined by real-time PCR and western blot, $p < 0.05$. (B) Upregulation of HOXB9 in co-cultured A549 cells validated by real-time PCR and Western blot, $p < 0.05$.

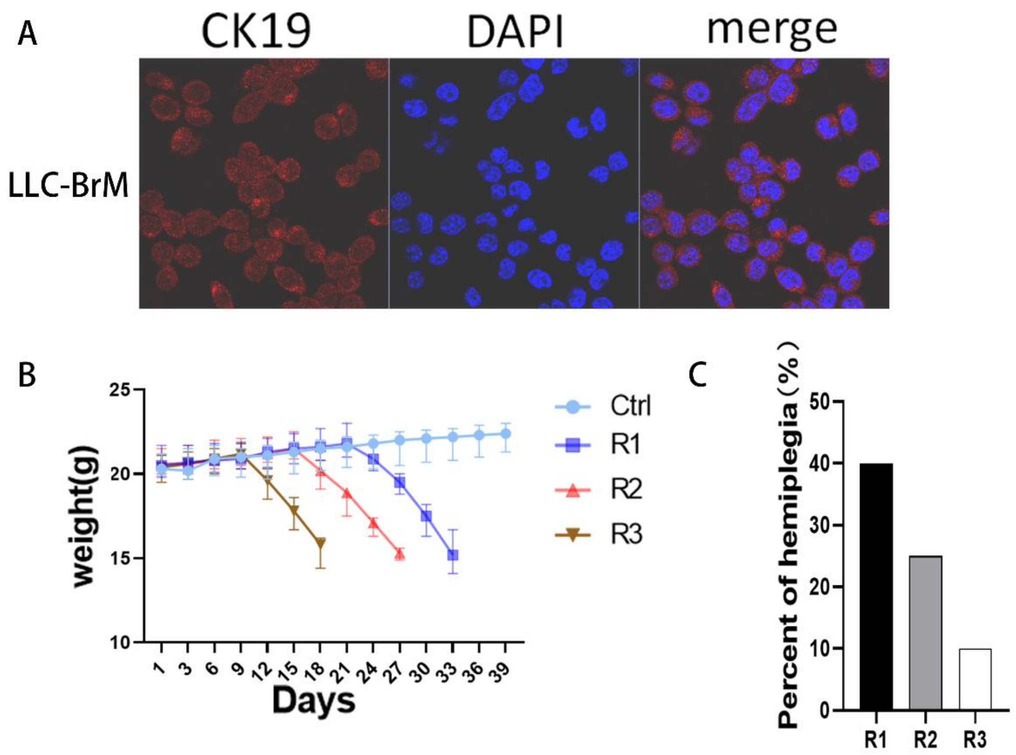

Supplementary Figure 4. Recorded physiological features of the model mice and determination of brain metastatic cells of LLC-BrM. (A) Determination of LLC-BrM with the marker of lung tumor cells, CK19. (B) Curves of the decreasing weights of the model mice. R1: mice in the first round of modeling; R2: mice in the second round of modeling; R3: experimental mice in the third round of modeling; Ctrl: the control group. (C) Record of hemiplegia percentage of the modeling mice in the three rounds of modeling.

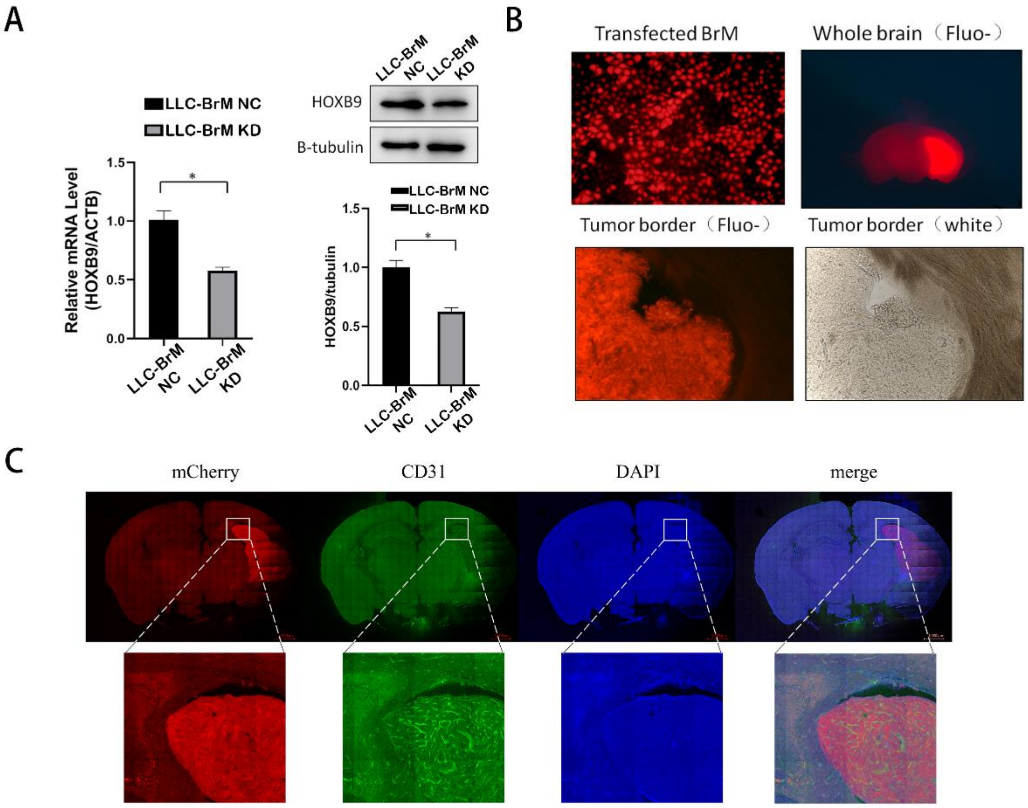

Supplementary Figure 5. Determination of HOXB9 expression knockdown and the brain metastases. (A) Determination of HOXB9 expression knockdown by real-time PCR and Western blot, $*p < 0.05$. (B) Determination of intracranial metastases by fluorescence microscope. (C) Determination of the brain metastases by two-photo scanning microscope.

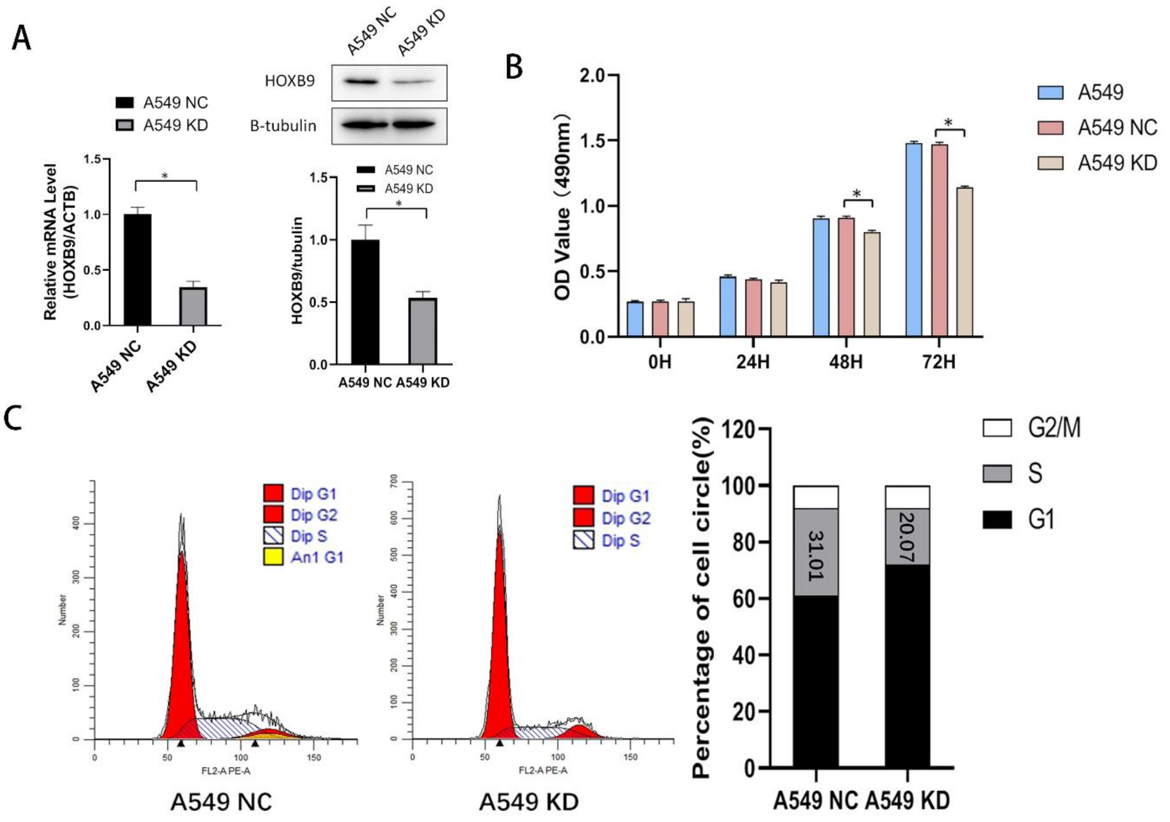

Supplementary Figure 6. Downregulation of HOXB9 induced the decreasing of the proliferation and S phase proportion of A549 tumor cells. (A) Determination of the HOXB9 expression knockdown by real-time PCR and Western blot, *$p < 0.05$. (B) Cell viability analysis of A549 cells with HOXB9 knockdown, *$p < 0.05$. (C) Flowcytometry analysis of the cell cycle of A549 cells with HOXB9 expression knockdown.

1.2 Supplementary Tables

Supplement table 1： NCI-H209 GSEA

Gene set name	NES	NOM p-val	FDR q-val
HALLMARK_HYPOXIA	2.114534	p<0.001	p<0.001
HALLMARK_MTORC1_SIGNALING	2.049872	p<0.001	p<0.001
HALLMARK_TNFA_SIGNALING_VIA_NFKB	2.03154	p<0.001	p<0.001
HALLMARK_INTERFERON_GAMMA_RESPONSE	1.963001	p<0.001	p<0.001
HALLMARK_INFLAMMATORY_RESPONSE	1.941752	p<0.001	p<0.001
HALLMARK_GLYCOLYSIS	1.935974	p<0.001	p<0.001
HALLMARK_IL2_STAT5_SIGNALING	1.932123	p<0.001	p<0.001
HALLMARK_ANDROGEN_RESPONSE	1.887269	p<0.001	p<0.001
HALLMARK_INTERFERON_ALPHA_RESPONSE	1.829837	p<0.001	p<0.001

Supplement Table 2: PC9 GSEA

Gene set name	NES	NOM p-val	FDR q-val
HALLMARK_HYPOXIA	1.7942404	P<0.01	P<0.01
HALLMARK_EPITHELIAL_MESENCHYMAL_TRANSITION	1.678764	P<0.01	P<0.01
HALLMARK_APICAL_JUNCTION	1.6677696	P<0.01	P<0.01
HALLMARK_TGF_BETA_SIGNALING	1.5673499	0.013	0.0291
HALLMARK_UV_RESPONSE_DN	1.5370948	0.007	0.0426

Supplement Table 3： LLC GSEA

Gene set name	NES	NOM p-val	FDR q-val
HALLMARK_E2F_TARGETS	2.607431	P<0.01	P<0.01
HALLMARK_MYC_TARGETS_V1	2.563842	P<0.01	P<0.01
HALLMARK_G2M_CHECKPOINT	2.40186	P<0.01	P<0.01
HALLMARK_OXIDATIVE_PHOSPHORYLATION	2.323324	P<0.01	P<0.01
HALLMARK_DNA_REPAIR	1.933659	P<0.01	P<0.01
HALLMARK_MYC_TARGETS_V2	1.884524	P<0.01	P<0.01
HALLMARK_REACTIVE_OXIGEN_SPECIES_PATHWAY	1.718278	P<0.01	P<0.01
HALLMARK_MTORC1_SIGNALING	1.51841	P<0.01	P<0.01
HALLMARK_FATTY_ACID_METABOLISM	1.491754	P<0.01	0.0198

Supplement Table 4. Statistics of the clinicopathological features of LUAD patients from TCGA database.

Clinical characteristics		Total （486）	%
Age at diagnosis (y)	age (<=60)	155	33.2
	age (>60)	312	66.8
Stage	I	262	54.8
	II	112	23.4
	III	79	16.5
	IV	25	5.2
T classification	T1	163	33.7
	T2	260	53.8
	T3	41	8.5
	T4	19	3.9
M classification	M0	333	93.3
	M1	24	6.7
N classification	N0	312	65.8
	N1	90	19
	N2	70	14.8
	N3	2	0.4
Gender	Male	222	45.7
	Female	264	54.3

Supplement Table 5: Cell-cycle-associated genes potentially transcribed by HOXB9 (Human)

symbol	start	end	score	strand	BindingSite
WEE2|NM_001105558.1	286	295	0.919998	-	TF=HOXB9;class=Homeo domain factors;sequence=GTCATAAAAA
CDC14A|XM_017002647.1	580	589	0.908943	-	TF=HOXB9;class=Homeo domain factors;sequence=CTCGTAAAAA
CDC14A|NM_033313.2	499	508	0.908943	-	TF=HOXB9;class=Homeo domain factors;sequence=CTCGTAAAAA
CDC14A|NM_001319212.1	499	508	0.908943	-	TF=HOXB9;class=Homeo domain factors;sequence=CTCGTAAAAA
CDC14A|NM_033312.2	499	508	0.908943	-	TF=HOXB9;class=Homeo domain factors;sequence=CTCGTAAAAA
CDC14A|NM_003672.3	499	508	0.908943	-	TF=HOXB9;class=Homeo domain factors;sequence=CTCGTAAAAA
CDC14A|NM_001319210.1	499	508	0.908943	-	TF=HOXB9;class=Homeo domain factors;sequence=CTCGTAAAAA
CHEK2|XM_017028561.2	791	800	0.887875	+	TF=HOXB9;class=Homeo domain factors;sequence=GTCCTAAAAA
ORC3|NM_001197259.1	117	126	0.887573	+	TF=HOXB9;class=Homeo domain factors;sequence=GTCTTAAAAA
ORC3|NM_012381.3	117	126	0.887573	+	TF=HOXB9;class=Homeo domain factors;sequence=GTCTTAAAAA
ORC3|NM_181837.2	117	126	0.887573	+	TF=HOXB9;class=Homeo domain factors;sequence=GTCTTAAAAA
ORC3|XM_011535652.3	56	65	0.887573	+	TF=HOXB9;class=Homeo domain factors;sequence=GTCTTAAAAA
ORC3|XR_001743295.2	56	65	0.887573	+	TF=HOXB9;class=Homeo domain factors;sequence=GTCTTAAAAA
ORC3|XR_001743294.2	56	65	0.887573	+	TF=HOXB9;class=Homeo domain factors;sequence=GTCTTAAAAA
ORC3|XM_017010635.2	56	65	0.887573	+	TF=HOXB9;class=Homeo domain factors;sequence=GTCTTAAAAA
ORC3|XM_011535651.2	56	65	0.887573	+	TF=HOXB9;class=Homeo domain factors;sequence=GTCTTAAAAA
ORC3|XM_017010634.2	56	65	0.887573	+	TF=HOXB9;class=Homeo domain factors;sequence=GTCTTAAAAA
ORC3|XM_005248704.2	56	65	0.887573	+	TF=HOXB9;class=Homeo domain factors;sequence=GTCTTAAAAA
ORC3|XM_017010633.2	56	65	0.887573	+	TF=HOXB9;class=Homeo domain factors;sequence=GTCTTAAAAA
ORC3|XM_017010632.2	56	65	0.887573	+	TF=HOXB9;class=Homeo domain factors;sequence=GTCTTAAAAA
ORC3|XM_017010640.1	48	57	0.887573	+	TF=HOXB9;class=Homeo domain factors;sequence=GTCTTAAAAA
ORC3|XM_017010637.1	48	57	0.887573	+	TF=HOXB9;class=Homeo domain factors;sequence=GTCTTAAAAA
ORC3|XM_017010639.1	48	57	0.887573	+	TF=HOXB9;class=Homeo domain factors;sequence=GTCTTAAAAA
ORC3|XM_005248706.1	48	57	0.887573	+	TF=HOXB9;class=Homeo domain factors;sequence=GTCTTAAAAA
ORC3|XM_017010636.2	43	52	0.887573	+	TF=HOXB9;class=Homeo domain factors;sequence=GTCTTAAAAA
ORC3|XM_017010638.2	43	52	0.887573	+	TF=HOXB9;class=Homeo domain factors;sequence=GTCTTAAAAA
ORC3|XM_005248705.2	43	52	0.887573	+	TF=HOXB9;class=Homeo domain factors;sequence=GTCTTAAAAA
SMAD3|NM_001145102.1	949	958	0.887573	-	TF=HOXB9;class=Homeo domain factors;sequence=GTCTTAAAAA
HDAC1|XM_011541309.2	715	724	0.880819	-	TF=HOXB9;class=Homeo domain factors;sequence=GTCGTGAAAA
HDAC1|NM_004964.2	711	720	0.880819	-	TF=HOXB9;class=Homeo domain factors;sequence=GTCGTGAAAA
CDC14B|NR_147239.1	81	90	0.874544	+	TF=HOXB9;class=Homeo domain factors;sequence=GTTGTAAAAT
CDC14B|NM_033331.3	81	90	0.874544	+	TF=HOXB9;class=Homeo domain factors;sequence=GTTGTAAAAT
CDC14B|NM_003671.4	81	90	0.874544	+	TF=HOXB9;class=Homeo domain factors;sequence=GTTGTAAAAT
CDC14B|NM_001351567.1	81	90	0.874544	+	TF=HOXB9;class=Homeo domain factors;sequence=GTTGTAAAAT
BUB3|NM_001007793.2	969	978	0.874544	+	TF=HOXB9;class=Homeo domain factors;sequence=GTGGTAAAAT
BUB3|NM_004725.3	969	978	0.874544	+	TF=HOXB9;class=Homeo domain factors;sequence=GTGGTAAAAT

SMAD3|NM_001145103.1	387	396	0.874544	-	TF=HOXB9;class=Homeo domain factors;sequence=GTGGTAAAAT
CDC14A|NM_033313.2	925	934	0.870194	+	TF=HOXB9;class=Homeo domain factors;sequence=GTCGTGAAAG
CDC14A|NM_001319212.1	925	934	0.870194	+	TF=HOXB9;class=Homeo domain factors;sequence=GTCGTGAAAG
CDC14A|NM_033312.2	925	934	0.870194	+	TF=HOXB9;class=Homeo domain factors;sequence=GTCGTGAAAG
CDC14A|NM_001319210.1	925	934	0.870194	+	TF=HOXB9;class=Homeo domain factors;sequence=GTCGTGAAAG
CDKN2B|NM_078487.2	107	116	0.8650	-	TF=HOXB9;class=Homeo domain factors;sequence=GTCATTAAAT
CDKN2B|NM_004936.3	107	116	0.865018	-	TF=HOXB9;class=Homeo domain factors;sequence=GTCATTAAAT
CCNB3|XM_017029914.1	244	253	0.864252	+	TF=HOXB9;class=Homeo domain factors;sequence=ATCATAAAAA
CCNA1|XM_011535295.2	86	95	0.862541	+	TF=HOXB9;class=Homeo domain factors;sequence=GTCATAAATC
CCNA1|XM_011535294.2	86	95	0.862541	+	TF=HOXB9;class=Homeo domain factors;sequence=GTCATAAATC
PKMYT1|XM_011522736.3	346	355	0.859748	-	TF=HOXB9;class=Homeo domain factors;sequence=GTCATAAATA
CDC14A|XM_024450506.1	500	509	0.857653	+	TF=HOXB9;class=Homeo domain factors;sequence=GTGGTAAAAC
SKP1|NM_006930.3	536	545	0.857653	+	TF=HOXB9;class=Homeo domain factors;sequence=GTAGTAAAAC
SKP1|NM_170679.2	536	545	0.857653	+	TF=HOXB9;class=Homeo domain factors;sequence=GTAGTAAAAC
CDKN2C|NM_001262.2	317	326	0.855605	+	TF=HOXB9;class=Homeo domain factors;sequence=TTCATAAAAT
CDC20|NM_001255.2	1	10	0.853627	-	TF=HOXB9;class=Homeo domain factors;sequence=ATCATAAAAG
E2F3|XM_005248865.5	550	559	0.851812	-	TF=HOXB9;class=Homeo domain factors;sequence=ATCCTAAAAT
E2F3|NM_001243076.2	487	496	0.851812	-	TF=HOXB9;class=Homeo domain factors;sequence=ATCCTAAAAT
RAD21|NM_006265.2	259	268	0.851812	-	TF=HOXB9;class=Homeo domain factors;sequence=ATCCTAAAAT
RAD21|NM_006265.2	506	515	0.85151	-	TF=HOXB9;class=Homeo domain factors;sequence=ATCTTAAAAT
CDC16|XM_017020833.1	14	23	0.85151	+	TF=HOXB9;class=Homeo domain factors;sequence=ATCTTAAAAT
DBF4|NM_001318061.1	8	17	0.851262	+	TF=HOXB9;class=Homeo domain factors;sequence=GTCATAAACA
DBF4|NM_001318060.1	8	17	0.851262	+	TF=HOXB9;class=Homeo domain factors;sequence=GTCATAAACA
DBF4|NM_001318062.1	8	17	0.851262	+	TF=HOXB9;class=Homeo domain factors;sequence=GTCATAAACA
DBF4|NM_006716.3	8	17	0.851262	+	TF=HOXB9;class=Homeo domain factors;sequence=GTCATAAACA

Only the genes with score larger than 0.85 were listed in the table.

Supplement Table 6： cell-cycle-associated genes potentially transcribed by Hoxb9 (Mouse)

symbol	start	end	score	strand	BindingSite
Ywhab|NM_018753.6	247	262	0.874794	+	TF=Hoxb9;class=Helix-Turn-Helix;sequence=GTAGTAATAAAATTTA
Stag1|XM_017313258.1	550	565	0.866441	+	TF=Hoxb9;class=Helix-Turn-Helix;sequence=ATAGTCATAAAAAAGG
Cdc20|NM_023223.2	10	25	0.866358	-	TF=Hoxb9;class=Helix-Turn-Helix;sequence=AAGGTCATAAAAAGAG
Pttg1|NM_013917.2	286	301	0.861438	+	TF=Hoxb9;class=Helix-Turn-Helix;sequence=AAAACCATAAAAGTGA
Pttg1|NM_001131054.1	553	568	0.861438	+	TF=Hoxb9;class=Helix-Turn-Helix;sequence=AAAACCATAAAAGTGA
Pttg1|XM_006533515.3	502	517	0.861438	+	TF=Hoxb9;class=Helix-Turn-Helix;sequence=AAAACCATAAAAGTGA
Pttg1|XM_006533516.3	515	530	0.861438	+	TF=Hoxb9;class=Helix-Turn-Helix;sequence=AAAACCATAAAAGTGA
Stag2|XM_011250984.2	217	232	0.860114	-	TF=Hoxb9;class=Helix-Turn-Helix;sequence=TGATCCATAAAAATGT
Stag2|NM_001077712.2	124	139	0.860114	-	TF=Hoxb9;class=Helix-Turn-Helix;sequence=TGATCCATAAAAATGT
Cdc14a|XM_017319563.1	941	956	0.859751	+	TF=Hoxb9;class=Helix-Turn-Helix;sequence=AAACTCATAAAATACC
Ccnd3|NM_007632.2	709	724	0.857922	+	TF=Hoxb9;class=Helix-Turn-Helix;sequence=AGAGCAATGAAATATG

Cdk2|NM_183417.3	132	147	0.855958	+	TF=Hoxb9;class=Helix-Turn-Helix;sequence=CCAGCAATAAAACAAC
Cdk2|NM_016756.4	132	147	0.855958	+	TF=Hoxb9;class=Helix-Turn-Helix;sequence=CCAGCAATAAAACAAC
Bub1b|XM_006498622.3	196	211	0.853704	-	TF=Hoxb9;class=Helix-Turn-Helix;sequence=CAGGCTATAAAAATAA
Bub1b|NM_009773.3	187	202	0.853704	-	TF=Hoxb9;class=Helix-Turn-Helix;sequence=CAGGCTATAAAAATAA
Cdc14b|XM_006517227.3	356	371	0.850623	+	TF=Hoxb9;class=Helix-Turn-Helix;sequence=AAAGCTATAAAAAGGC
Cdc14b|XR_873438.2	356	371	0.850623	+	TF=Hoxb9;class=Helix-Turn-Helix;sequence=AAAGCTATAAAAAGGC

Only gene with score larger than 0.85 were listed in the table.
